# Effect of core strength training on the badminton player’s performance: A systematic review & meta-analysis

**DOI:** 10.1371/journal.pone.0305116

**Published:** 2024-06-12

**Authors:** Shuzhen Ma, Kim Geok Soh, Salimah Binti Japar, Chunqing Liu, Shengyao Luo, Yiqiang Mai, Xinzhi Wang, Mengze Zhai

**Affiliations:** 1 Department of Sports Studies, Faculty of Educational Studies, Universiti Putra Malaysia, Selangor, Malaysia; 2 School of Public Administration, Guilin University of Technology, Guilin, China; 3 School of Physical Education and Arts, Jiangxi University of Science and Technology, Ganzhou, China; 4 Physical Education Department, Tianjin Binhai Automotive Engineering Vocational College, Tianjin, China; Ningbo University, CHINA

## Abstract

**Background:**

Core strength training (CST) has been shown to improve performance in several sports disciplines. CST is recognized as one of the crucial elements that enhance athletic performance, particularly impacting badminton skills. Despite its popularity as a strength training method among badminton players, there is a lack of comprehensive studies examining the effectiveness of CST on the performance of these athletes.

**Objective:**

This study aims to ascertain CST’s effects on badminton players’ performance.

**Method:**

This study followed PRISMA principles and conducted comprehensive searches in well-known academic databases (SCOPUS, Pubmed, CNKI, Web of Science, Core Collection, and EBSCOhost) up to August 2023. The inclusive criteria were established using the PICOS framework. Following their inclusion based on PICOS criteria, the selected studies underwent literature review and meta-analysis. The methodological quality of the assessments was evaluated using Cochrane Collaboration’s risk of bias tools bias risk tools and recommendations for a graded assessment, development, and evaluation.

**Results:**

The analysis included participants aged 10–19 years from 13 studies of moderate quality, totaling 208 individuals. The CST intervention s lasted between 4 to 16 weeks, with a frequency of 1 to 4 sessions per week and each session lasting 20 to 120 minutes. Sample sizes across these studies ranged from 8 to 34 participants. According to the meta-analysis, CST significantly influenced badminton performance, particularly in areas of explosive power (ES = 0.03 P = 0.04), front-court skill (ES = 2.53, P = 0.003), and back-court skill (ES = 2.33, P = 0.002).

**Conclusion:**

CST enhances badminton players’ fitness (strength, power, balance, and stability), in situ (front/back-court) skills, and movement position hitting. However, its effects on speed, endurance, agility, flexibility, and coordination are unclear, revealing a research gap. The precise benefits of CST, especially on flexibility and specific hitting skills (smashes, clears, drives, net shots, crosscourt, push, and lift shots), need more investigation. Additionally, research on CST’s impact on female athletes is significantly lacking.

## Introduction

With frequent starts, stops, jumps, leaps, lunges, and quick direction changes, badminton is recognized as the fastest racquet sport in the world [1]. A wide range of deft postural adjustments and motions are necessary for badminton [2]. To become an elite-level athlete, a high level of skill is required [3, 4]. The movement of badminton players when hitting the ball can be categorized into in situ skills and movement position hitting skills [5]. Based on the various court areas, the performance of badminton skills is divided into three categories: front court (such as spinning net shots, lift shots, crosscourt shots, push shots, and rush shots), mid court (such as drives), and backcourt (such as smashes, drop shots, and hight clears) [6].

Over the past few decades, there has been a rise in badminton research, which has updated our understanding of the physical, biomechanical, psychological, tactical, technical, and timing aspects of players [7, 8]. Researchers have discovered that athletes’ physical health and skills influence their competitive capacity [9]. Only if players maintain robust physical ability can they handle emergencies on the court and secure victories [10]. Physical fitness in badminton encompasses muscular strength, power, speed, coordination, endurance, flexibility, agility, stability, and balance [11–13]. In badminton, maintaining shuttle stability and controlling stroke trajectory are crucial. The core strength focus is on stabilizing the players’ core body parts by harnessing their arms and legs’ power to control the shuttle’s formation and movement trajectory. Thus, it is vital to examine how players’ core strength impacts their movement during badminton play [14].

In the 1990s, core strength training (CST)—a novel concept in strength training- was introduced to rehabilitate competitive athletes [15]. The competitive sports training community in China has increasingly come to accept and value CST in the 21st century. Thus far, CST has emerged as a particularly significant and unique form of strength training, gaining popularity among athletes [16]. A strong core serves as the foundation for the muscles of the upper and lower limbs, facilitating the acceleration of body parts during the performance of motor skills and the transfer of power between proximal and distal body parts [3, 17]. Core stability training is essential for any racket sports player, but it is especially critical for badminton players who frequently perform smashes throughout the game [18]. Fitness experts are growing concerned about utilizing trunk stability exercises [19]. In recent years, CST has gained popularity as a means to enhance performance [10]. However, further research is necessary to understand the benefits of CST for professional athletes and to determine the most effective ways to conduct such training to maximize athletic performance [20].

Numerous studies suggest that the core muscles facilitate proximal stability for distal mobility [17, 21–23]. In sports, core stability optimally stabilizes the torso’s position and motion, enabling the efficient transfer and regulation of force from the body’s center to the limbs [24]. The core area acts as a hinge or bridge that connects the upper and lower limbs, facilitating movement. The stability of this link not only determines the firmness of the limbs’ fulcrum but also influences the accuracy and quality of overall body movements [25–27]. For athletes, core training should be tailored to the necessary sports skills [28]. Badminton players need a sufficiently strong core and high dynamic balance to execute rapid postural changes on the court [29].

The core is the foundation or engine of all limb movements, known in alternative medicine as the "powerhouse" [3, 29, 30]. Historically, core stability exercises frequently treated lower extremity and lower back injuries [31]. Core stability training has recently been shown to enhance player performance [31]. Athletes’ performance is directly impacted by their ability to balance, jump vertically, move quickly, and compete [32–34]. Athletes’ ability to compete, balance, vertical leap height, and movement speed are crucial to their performance. For instance, after a spike shot, volleyball players frequently lose their balance and change their center of gravity, landing on one leg. Such single-leg landings following a volleyball spike may increase the risk of anterior cruciate ligament injury more than landing on both legs [35]. In badminton, the development of badminton-specific footwork training has evolved from traditional physical exercises to novel intervention approaches [36], where good balance significantly improves players’ on-court footwork performance [2]. An excellent vertical leap height may make the players smash more effectively [37], and a rapid moving speed can make the badminton players more active when receiving the opponent’s return ball, boosting badminton players’ chances of winning the match [38]. Possessing a solid competitive ability can significantly enhance athletes’ chances of winning [39].

For athletes to improve performance, core exercises must be more intricate and demanding [9]. Core training is the entire program’s foundation and must be combined with static low-intensity core stability training and high-load dynamic CST [9]. The two main categories of core training are static and dynamic CST [40]. In static CST, the joint and muscle are either held in a static position while being opposed by resistance (sub-maximal muscle action) or working against an immovable force (maximal muscle action) [41]. Meanwhile, dynamic CST can be regarded as dynamic exercises performed to exert a muscle force concentrically, eccentrically, repeatedly, or continuously over time [40].

Moreover, the surface of the core exercise can also be changed in dynamic CST to promote higher proprioceptive demands and, consequently, increased core activation [42]. In addition, if athletes neglect CST, their ability to control and use muscle strength throughout the body will be impaired, potentially increasing their risk of sports injuries [9, 43]. Studies suggest that weak or uncoordinated core muscles disrupt energy transfer. Strain and overuse also reduce movement effectiveness, which could result in injuries [44, 45]. Kimura also found that when badminton players landed with a single leg after overhead stroke skills, their knee valgus moment increased due to inadequate trunk flexion strength [34]. Therefore, exercises for core stability are commonly utilized to prevent lower back and lower limb injuries, and athletes who neglect their core suffer injuries [32, 33, 34].

In badminton, the stability of the ball’s return is crucial due to various uncertain factors such as angle, arc distance, and ball direction [16]. The core musculature serves as a connecting bridge between the upper and lower extremities, playing a crucial role in transporting energy from the proximal to the distal body segments [46, 47]. This stabilization helps regulate the center of gravity and connects the upper and lower limbs, forming the basis for badminton players to execute techniques such as sprinting, throwing, and jumping [17]. Consequently, core strength plays a crucial role as the fundamental component in badminton movements [16], and CST helps to improve badminton techniques. Research has demonstrated CST’s significant impact on enhancing badminton performance [48]. Rotational trunk strength is essential for badminton players because it affects how the body moves during the smashing part of the smash skill. A strong core helps badminton players maintain a steady stroke and improve their smashing technique [49].

Athletes in football, handball, basketball, swimming, dancing, Karate, Muay Thai, gymnastics, volleyball, badminton, and golf can all benefit from CST [9]. For example, prior research has demonstrated that golfers’ repeated club-head speed and backspin assessments slightly decreased variability following CST (-8.2%), suggesting a more stable golf swing [50]. Following CST, karate athletes demonstrated a significant change in post-test results for the spinning wheel kick evaluation compared to the control groups [51]. Additional data is required, such as from running and volleyball, to validate the impact of CST on badminton performance. This includes explaining the differences in gait patterns between high and low-mileage runners using machine learning [52, 53] and examining temporal kinematic and kinetic differences in various landing techniques following volleyball spike shots [35, 54]. This review and meta-analysis aim to provide an extensive overview of how CST affects badminton players’ performance.

## Methods

### Protocol and registration

This systematic review and meta-analysis was conducted in adherence to the PRISMA statement [55], as registered on Inplasy.com (INPLASY2023110098).

### Search strategy

This study utilizes well-known databases to search for relevant literature, including SCOPUS, PubMed, CNKI, Web of Science Core Collection, and EBSCOhost, up until September 2023. The search keywords employed are: (“Core strength training” OR “Core training” OR “Core-muscle training” OR “Core exercise” OR “Core-stability exercise”) AND (“Badminton athletes” OR “badminton players” OR “badminton beginners” OR “shuttler”).

### Criteria

The PICOS criteria, including population, intervention, comparison, outcome, and study design, are detailed in Table 1. The primary focus of this study is how badminton players’ performance is affected by core strength training (CST). The study will include the literature if it satisfies the following requirements.

1) Population: The test objects are badminton players of different skill levels. The skill level of badminton players is divided from low to high, ranging from beginners, school level, provincial level, and national level.

2) Intervention: The intervention involves conducting CST experiments for a duration of more than four weeks, as studies have shown that training periods shorter than four weeks do not significantly enhance athletes’ performance [56, 57].

3) Comparison: At least two groups were included, and the CST group was compared to other training or no training as the comparison benchmark.

4) Outcome: The outcome was centered on the badminton players’ performance. This study primarily focuses on badminton performance’s physical fitness and skill aspects. The definition of skills performance is as follows. Based on the various court areas, badminton skills performance is divided into three categories: front court (such as spinning net shot, lift shot, crosscourt shot, push shot, and rush shot), mid court (such as drive), and backcourt (such as smash, drop shot, and hight clear) [6]. Whether badminton players need to move when hitting the ball can be divided into situ skills and move position hit skills [5]. On the other hand, some components of badminton physical fitness performance are muscular strength, power, speed, coordination, endurance, flexibility, agility, stability, and balance [11–13].

5) Study design: This review considers randomized controlled studies.

**Table 1:** Inclusion criteria according to the PICOS conditions.

Items	Detailed inclusion criteria
Population	Badminton athletes
Intervention	Core training (not less than 4 week)
Comparison	Two or more groups
Outcome	Badminton player’s performance
Study designs	RCT

### Study selection

This review and meta-analysis initially utilize the EndNote citation management system to eliminate duplicate research papers. Two impartial reviewers assess the titles and abstracts of articles, selecting those that meet the predetermined inclusion criteria and rejecting those that do not. Articles chosen in this manner are then read in full. During this phase, any articles that cannot be accessed in full text will be excluded. In cases of differing opinions, a third reviewer is consulted to provide advice until a consensus is achieved.

### Data extraction and quality assessment

After completing the data search, data from qualified studies were collected using a predetermined extraction form that included: 1) author name and publication year, 2) type of athletes, 3) population characteristics, 4) study design (including week, frequency, whether randomized, methods, and intensity), 5) measures and intensity, and 6) outcomes. The PEDro scale, known for its strong validity and reliability, has been demonstrated to be a dependable indicator of methodological quality in constructing a systematic review.

In systematic reviews, the PEDro scale is frequently employed [58]. It has demonstrated excellent validity and reliability, highlighting the advantages of using the PEDro scale as a valuable tool for evaluating the quality of experimental methodology [59]. The purpose of the PEDro scale is to assess four main methodological components of a study: data analysis, group comparison, blinding procedures, and randomization processes [60]. The scale comprises 11 parts: 1) inclusion criteria and source; 2) random allocation; 3) allocation concealment; 4) baseline comparability; 5) blinding of subjects; 6) blinding of therapists; 7) blinding of assessors; 8) adequate (>85%) follow-up; 9) intention-to-treat analysis; 10) between-group comparison; and 11) point estimates and variability [61]. An overall score, ranging from 0 to 10, is calculated by adding the answers for items 2 through 11, as item 1, which addresses external validity, is not included in the grading [61].

The assessment of the 11 items on the PEDro scale was carried out by two independent raters using ‘yes’ (1 point) or ‘no’ (0 points). Furthermore, a third rater resolved any disagreements that arose during the scoring process. The quality of the methodology improves with higher PEDro scores. It is important to note that the eligibility criteria score is not included in the final score due to its correlation with external effectiveness. Research achieving a score of 8 to 10 is considered methodologically outstanding. Studies with a score of 5 to 7 are regarded as good, those scoring 3 to 4 are fair, and studies scoring less than 3 are considered poor [62]. The reliability of the PEDro total score is passably excellent [63, 64], although the dependability of individual scale items varies from moderate to outstanding [63, 65–67]. Therefore, this study primarily focuses on evaluating article quality based on the total PEDro score, which ranges from 0 to 10, where higher scores indicate better methodological quality [60].

### Risk of bias

The Cochrane Risk of Bias (RoB) tool was utilized to conduct the RoB assessment [68]. It assesses RoB across seven domains: random sequence generation, concealment of assignment, blinding of participants and personnel, blinding of outcome assessment, incomplete outcome data, selective reporting, and other sources of bias [69]. Each domain can be categorized as ‘low’, ‘unclear’ or ‘high’ risk. Following guidelines on the Cochrane Training webpage, two reviewers independently used the most recent version of the Cochrane RoB assessment for randomized trials (RoB-2) to evaluate the RoB of each identified RCT [70].

### Statistical analysis

Although the meta-analytical comparison required only two studies [71], the sample size for CST is relatively small. Therefore, we only conducted a meta-analysis when three or more studies reported data on the above technical skills outcomes [72]. To reflect the effect size (ES), we used the mean and standard deviation of the performance indicators before and after the intervention. We then normalized the data using the post-intervention performance measures [73]. The meta-analysis employed the random-effects mode [74], facilitating analysis while accounting for heterogeneity across studies [75]. The I^2^ statistics were used to assess the impact of study heterogeneity, with values of < 25%, 25–75%, and > 75% indicating low, moderate, and high levels of heterogeneity, respectively [76]. The extended Egger’s test was used to investigate the risk of publication bias [77, 78], with a statistical significance threshold of P < 0.05.

## Results

### Study selection

**Fig 1:**
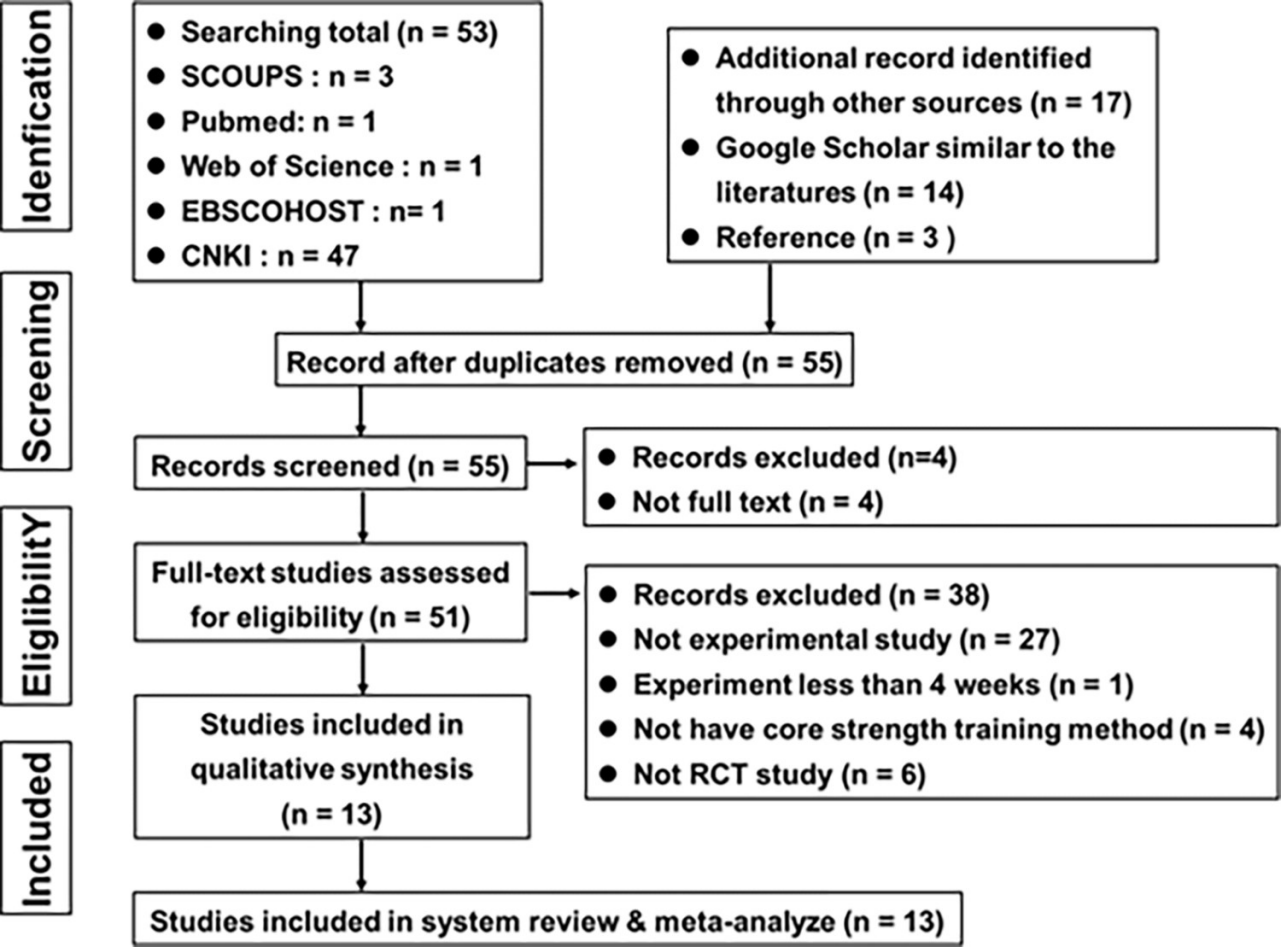
PRISMA flow chart of the study selection process.

The record selection flow chart is depicted in Fig 1. This study retrieved 70 publications from databases, including grey literature. Of these, 53 were sourced from five main databases: 3 from SCOPUS, 1 from PubMed, 1 from the Web of Science Core Collection, 1 from EBSCOhost, and 47 from CNKI. After removing duplicates, 55 papers were identified as research articles, of which 51 full-text studies were subsequently evaluated in the next phase, following the disqualification of 4 full-text articles for reasons not specified at this point. In the elimination phase, 27 articles were excluded for not being experimental studies, 1 for having an experimental duration of less than 4 weeks, 4 for lacking core strength training methods, and 6 for not being RCT studies. Ultimately, the analysis included a total of 13 publications.

### Risk of bias

**Fig 2:**
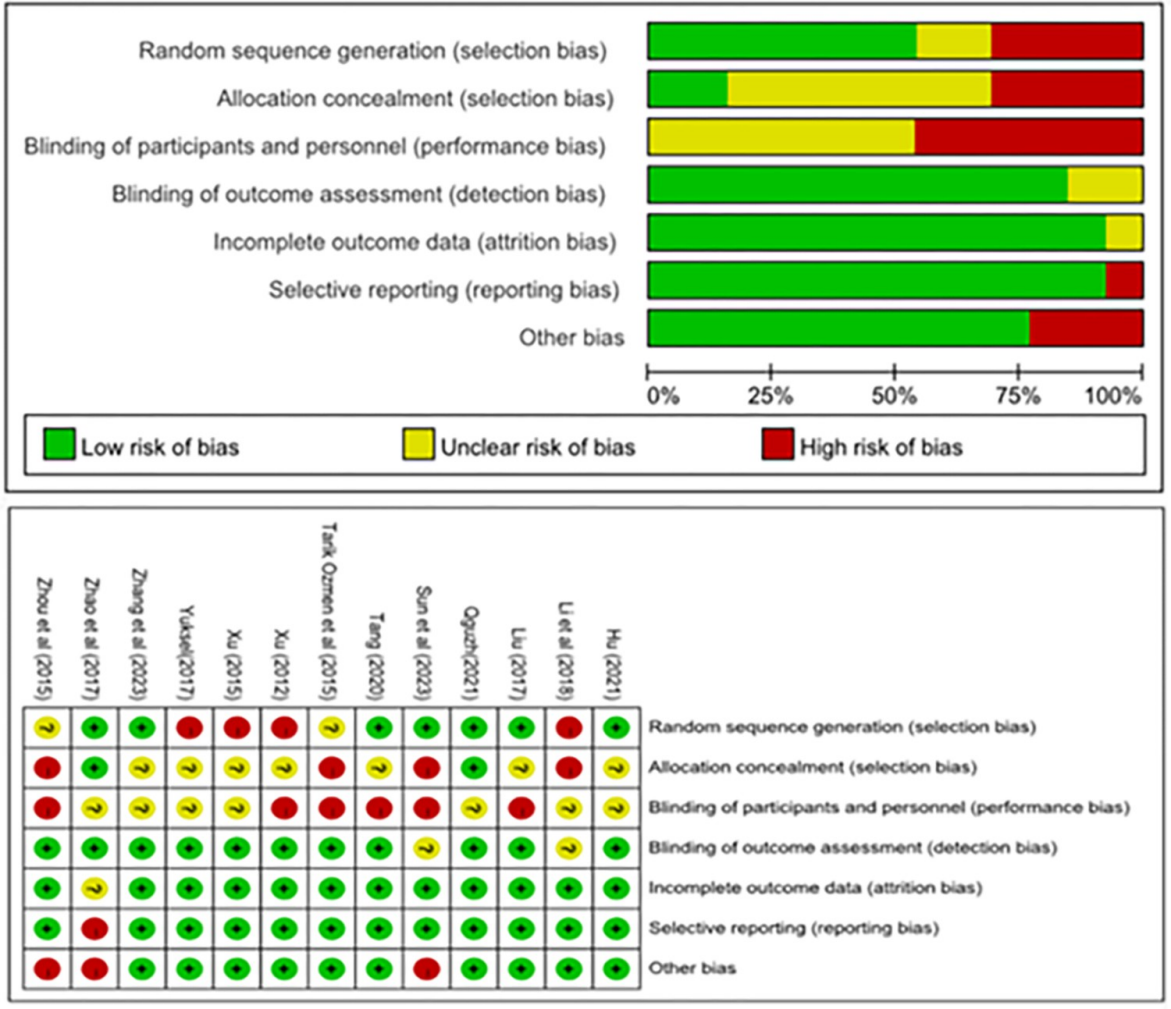
Risk of bias graph and summary.

In this graph (Fig 2), each row represents a study, and each column corresponds to a type of bias. The color denotes the likelihood of each bias type occurring in the studies: green for a low risk of bias, yellow for an unclear risk of bias, and red for a high risk of bias (RoB). The analysis identified high RoB in random sequence generation (selection bias), allocation concealment (selection bias), and blinding of participants and personnel (performance bias) across these 13 studies. Among these, blinding of participants and personnel was found to pose the highest risk. Conversely, blinding of outcome assessment (detection bias), incomplete outcome data (attrition bias), selective reporting (reporting bias), and other biases were associated with a lower risk.

### Participant characteristics

The population characteristics of the 13 included studies (Table 3) were reported based on the following criteria: (1) Geographical location: One study was from Turkey [79], three from India [79–81], with the remainder originating from China. (2) Sample size: The studies involved a total of 208 subjects, averaging 16 participants each, ranging from 8 to 28. (3) Gender: In this study, 6 articles focused on males [27, 82–86], 4 articles focused on females combined with males [80, 81, 87, 88], and the remaining 3 articles did not specialize on gender [79, 89, 90], no articles focused on females. (4) Level: 10 articles had participants at the school level [27, 81–87, 89, 90], 1 article included participants at the beginner level [79], 1 article contained participants at the provincial level [88], and 1 article involved participants at the national level [86]. (5) Age: Participants’ ages spanned from 10 to 29 years, with a focus on the 18–22 year age group in the review articles, except one which did not specify an age range [81]. (6) Body Mass Index (BMI): The subjects’ heights and weights were reported as follows—males: 1.72–1.80 m in height and 57.58–75 kg in weight; females: 1.68–1.70 m in height and 49.82–50.75 kg in weight. The BMI for men was primarily within 18.3–23.2, and for women, it was 17.5–17.6. Two articles did not report BMI or specify gender [80, 81].

### PEDro score and study design

The PEDro scores of the included articles are summarized in Table 2, with scores ranging from 3 to 6. The mean PEDro score was 4.61, indicative of moderate quality, with overall reliability assessed as ranging from ’fair’ to ’good’ [63]. Score differences primarily depended on the inclusion of group comparisons. The study designs are detailed in Table 3, with all articles being published between 2012 and 2023. The duration of interventions reported was between 4 to 16 weeks, with the shortest and longest durations being 4 weeks and 16 weeks, respectively. Of the studies, 11 reported the frequency of interventions, whereas two did not specify this detail [83, 88]. Additionally, 11 studies employed a randomized design, while two did not use random assignment for participant groups [83, 87].

**Table 2:** Population, study design PEDro scale.

Study	Eligibility criteria	Random allocation	Allocation concealment	Baseline comparability	Blind participants	Blind assessor	Blind Therapist	Follow-up	Intention to treat analysis	Between group comparisons	Point measure variability	Total PEDro Score
Sun et al. (2023)	1	1	0	1	0	0	0	1	0	0	1	4
Tarik et al. (2015)	1	1	0	1	0	0	0	1	0	1	1	5
Li et al. (2018)	1	1	0	1	0	0	0	1	0	1	1	5
Liu (2017)	1	1	0	1	0	0	0	1	0	1	1	5
Zhang et al. (2023)	1	1	0	1	0	0	0	1	0	1	1	5
Hu (2021)	1	1	0	1	0	0	0	1	0	1	1	5
Xu (2015)	1	0	0	1	0	0	0	1	0	1	1	4
Zhou et al. (2015)	1	1	0	1	0	0	0	1	0	0	1	4
Tang (2020)	1	1	0	1	0	0	0	1	0	1	0	4
Xu (2012)	1	0	0	1	0	0	0	1	0	1	1	4
Zhao et al. (2017)	1	1	0	1	0	0	0	1	0	0	1	4
Oğu et al. (2021)	1	1	0	1	0	0	0	1	0	0	1	5
Mod et al. (2017)	1	1	0	1	0	0	0	1	0	1	1	6
Total	13	11	0	13	0	0	0	13	0	9	9	60

Note: A detailed explanation for each PEDro scale item can be accessed at https://www.pedro.org.au/english/downloads/pedro-scale [73]. *From a possible maximal punctuation of 10.

**Table 3:** Characteristics of studies examined in the present review & meta-analysis.

Study	Population		Intervention		Measures index	Outcome
	Type	Characteristics (y/m/kg)	(wk/f/min)	Method			Randomized
Sun et al. (2023)	school level	EG: 10(M) = 18.2±0.7/ 175.2±2.4/66.1±3.2 CG: 10(M) = 17.8±1.3 /174.7±1.5/67.0±3.4	EG = 10/3/25 CG = 10/3/25	EG: CCST CG: NT	Yes	FMS scores (scores, hurdles, straight squat shoulder flexibility, Lower waist flexibility, body control push up swivel stability) -------------- Skill Test (back toss medicine ball, badminton throw, Forehand kick, Backhand draw, Forehand kick, backhand kick)	Straight squat (p < 0.05)↑, Swivel stability (p < 0.05)↑ OT ↔ ----------- Badminton throw (p < 0.01)↑ Backhand draw, Forehand kick, Backhand kick (p < 0.05)↑ OT ↔
Tarik et al. (2015)	beginners	EG/CG: 5(N) = 10.8 ± 0.3/140.6 ± 4.4/33.9 ± 5.8	EG = 6/2/N CG = 6/2/N	EG: CCST CG: NT	Yes	physical fitness (Agility, directions)	Directions of SEBT and core endurance tests (p < 0.05)↑
Li et al. (2018)	school level	EG: 10(N) = 22.5±0.5 /179.1±4.1/ 72.3±5.4 CG: 10(N) = 22.8±0.4/ 179.5±5.2/63.62±6.08	EG = 8/2/N CG = 8/2/N	EG: CCST CG: TST	Yes	Physical fitness (Run with straight back, 30 second double shake rope skipping, standing long jump, low center of gravity corner running) ------------ Skill Test: Hitting techniques	Low center of gravity corner running (P < 0.05)↑ OT↔ ----------- Hitting techniques (P < 0.01)↑
Liu (2017)	school level	EG: 13(N) = 20.85±0.99/ 172.15±4.85/72.3±5.4 CG: 13(N) = 20.92±1.38 /172.92±4.61/63.62±4.17	EG = 8/4/120 CG = 8/4/120	EG: CCST CG: TST	Yes	Physical fitness (Standing shuttlecock、One minute sit-ups, Standing broad jump, 25*5 Shuttle run, low center of gravity corner running) ------------ Skill Test (Forehand to a high backcourt shot, forehand chop, Forehand kill, net kill, Scrub the ball in front of the net)	Standing shuttlecock (P < 0.01)↑, Low center of gravity corner running (P < 0.05)↑ OT ↔ ------------ Forehand to a high backcourt shot (P < 0.05)↑, Forehand kill (P < 0.01)↑, Net kill (P < 0.01)↑ OT ↔
Zhang et al. (2023)	provincial level	EG: 10(F/M) M: 5 = 17.4±0.7/ 178.15±1.88/58.62±1.61 F: 5 = 17.5±1.0/ 168.13±2.11/49.82±1.68 CG: 10(F/M) M: 5 = 17.2±1.11/ 177.45±2.69/57.58±2.57 F: 5/ = 17.7±0.8/ 170.20±2.56/ 50.75±2.10	EG = 12/N/N CG = 12/N/N	EG = SCST CG = DCST	Yes	Physical fitness (Core muscle strength 60/(°)、180/(°)) ------------ BFMC stability test (Angle/(°): -45/(°), -90/(°), -135/(°), 180/(°), 135/(°), 90/(°), 45/(°), 0/(°))	CG: 60/(°), Maximum torque of torso to the left and torso to the right↑ 180/(°), Average power, maximum torque, and maximum power in constant velocity data↑ ------------ CG: -45/(°), -135/(°), 135/(°), 45/(°) ↑ DG: -90/(°), 180/(°), 90/(°), 45/(°), 0/(°). ↑
Hu (2021)	school level	EG: 14(F/M) M: 11/ F:3 = 24.00±1.18/ 174.00±7.99/68.21±12.47 CG: 14(F/M) M: 11/ F: 3 = 23.36±1.01/ 174.21±7.14/67.57±11.19	EG = 8/2/N CG = 8/2/N	EG: CCST CG: NT	Not	Skill Test (High ball technique) ------------ Psychological index (Confidence score, Volitional quality score)	High ball technique (P < 0.05)↑ ----------- Confidence score (P < 0.05)↑ Volitional quality score (P < 0.001)↑
Xu (2015)	school level	EG: 10(M) = 21.5/173/67.3 EG: 10(M) = 21.8/172/65.7	EG = 12/3/80 CG = 12/3/80	EG: CCST CG: TST	Yes	Physical fitness (Run 40 meters quickly, 30 second double shake rope skipping, 60 seconds push-ups, standing long jump, About 5 round trips, Plank) ------------- Skill Test (lift shot/spinning net shot, Crosscourt shot, Push shot, Net Lift, Total score)	30 second double shake rope skipping, About five round trips (P < 0.05)↑, Plank (P < 0.01)↑ OT ↔ ------------ Crosscourt shot (P < 0.01)↑ Net lift, Total score (P < 0.05)↑
Zhou et al. (2015)	school level	EG: 8(M) = 21±1/ 174.40±2.67/66.20±6.35 CG: 8(M) = 21±1/ 173.50±3.30/65.70±6.35	EG = 4/3/N CG = 4/3/N	EG: CCST CG: TST	Yes	Physical fitness (Field moving speed)	Field moving speed (P < 0.05)↑
Tang (2020)	school level	EG: 4(M) = 22/177.4/68.8 CG: 4(M) = 22/176.2/67.1	EG = 8/2/N CG = 8/2/N	EG: CCST CG: TST	Yes	Specific physical fitness (Linear high net, Full-court pace, Take off and kill the ball, Two high hang in the back (pass rate))	Test all indicators↑
Xu (2012)	school level	EG: 10(M) = 27.70±1.160/ 179.80±4.917/75.00±6.782 EG: 10(M) = 27.40±1.430/ 179.30±5.982/73.50±5.968	EG = 16/N/N CG = 16/N/N	EG: CCST CG: TST	Not	Skill Test (Batting technical index) ------------ Physical fitness: 30 meter start run, 30 seconds skipping rope, Throw solid ball in place, Put the shot put sideways in place, Standing long jump, 25m x 5 round trip run)	Batting technical index (P < 0.01)↑ ------------ 30 meter start run, 30 seconds skipping rope, Throw solid ball in place, Put the shot put sideways in place, Standing long jump (P < 0.05)↑ OT ↔
Zhao et al. (2017)	school level	EG: 8(M) = 18.25±0.71 /175.19±2.37/66.13±3.21 CG: 8(M) = 17.75±1.28/ 174.65±1.48/67.01±3.38	EG = 10/3/25 CG = 10/3/25	EG: CCST CG: TST	Yes	Physical fitness (FMS score, Squat, hurdle, straight squat, shoulder flexibility, low back flexibility, control body push-up, rotation stability) ------------ Special Skill Test (badminton toos, forehand draw (standard), backhand draw (standard), Forehand draw (Technical assessment), backhand draw (technical assessment))	Straight squat, Rotation stability (P < 0.05)↑ OT ↔ ------------- Badminton toss (P < 0.01)↑, backhand draw (standard), forehand draw (Technical assessment), backhand draw (technical assessment) (P < 0.05)↑ OT ↔
Modified et al. (2017)	national level	EG/CG: 10(M/F) = 18.98±1.92/N/N	EG = 8/3/20-25 CG = 8/3/20-25	EG: CCST CG: NT	Yes	Star Excursion Balance Test	Dynamic balance↑
Oğuzhan Yüksel et al. (2021)	school level	EG/CG: 17(M/F) = N/N/N	EG = 6/2/N CG = 6/2/N	EG: CCST CG: PT	Yes	Star Excursion Balance Test (SEBT), Core Muscle Endurance Test (CMET), Illinois Agility Test (IAT). Results	Equally effective in promoting dynamic balance, core endurance and agility

EG, Experimental group; CG, Control Group; M, Male; F, Female; CST, CST; CCST, Dynamic and static combined with CST; SCST, Static CST; DCST, Dynamic CST; PT, Plyometric training; NT, Not training; TST, Traditional strength training; N, Not; significant improvement; ↓, significant decrease; ↔, no significant difference; Wk, week; f, frequency; min, minute; -------, Test the dividing lines of multiple metrics.

### Training programs

Table 3 details the intervention types across 13 studies, focusing on methods, duration, frequency, and session length. The studies showed consistency with prior research in subject levels and laboratory settings. The objective was to identify discrepancies or confirm consistency in experimental controls. The majority utilized both dynamic and static training methods, with experiments typically dividing participants into two groups for comparison. Most studies contrasted the core strength training (CST) group with the traditional strength training (TST) group over an eight-week period, training 2–3 times weekly, though training intensity was not specified. Specifically, 12 articles reported combining dynamic and static training under CST [27, 79–87, 89, 90], with one study comparing dynamic to static core strength [88]. To underline training effects, 4 studies included a non-training (NT) control group [27, 79, 80, 87], 7 compared CST to TST [82–86, 89, 90], one contrasted it with plyometric training (PT) [80], and one study directly compared dynamic core strength (DCS) to static core strength (SCS) [88]. All researchers divided participants into two groups for controlled comparisons.

On the other hand, while 13 authors applied the randomized method to ensure experimental randomness, the methodologies of two other authors require clearer elaboration [83, 87]. RCTs enable the management of several forms of bias that are difficult to control in other study designs, such as cohort, case-control, and non-randomized controlled trials [91]. Thus, this study advocates for the use of RCTs due to their significant contributions to conducting research with high relevance and validity. (1) Intervention Period: Regarding the intervention duration, frequency, and session length, two articles reported training sessions of 25 minutes each [27, 82], and one varied between 20–25 minutes [80]. Other specified durations included 80 minutes and 120 minutes [81, 86, 89]. Eight articles did not detail training intensity [79, 81, 83–85, 87, 88, 90]. (2) Intervention Duration: Training durations spanned from 4 to 16 weeks. Five studies favored an 8-week regimen, the most common duration [80, 84, 87, 89, 90]. Additional durations included 4 weeks [85], 6 weeks [79, 89], 10 weeks [27, 82], and 12 weeks [86, 88], with one study extending to 16 weeks [83]. (3) Intervention Frequency: Sessions ranged from 2 to 5 times weekly. Five studies scheduled training twice a week [79, 81, 84, 87, 90], another five three times a week [27, 81, 82, 85, 86], and one four times weekly [89]. Two studies’ frequency details were lacking [83, 88].

### Meta-analysis results and outcome

Among the 13 articles included in this review’s meta-analysis, 11 examined the impact of CST on the physical fitness of badminton players [27, 79–83, 85, 86, 88–90], 8 explored the impact of CST on the technical quality of badminton players [27, 79, 82–84, 86, 87, 89], and only 1 article addressed the impact of CST on the psychological aspects of badminton players [87]. There is no consensus among the more frequently studied indicators. Therefore, a meta-analysis of metrics from three or more studies, which were inconsistent, was conducted to find a more precise answer.

### Effect of core strength training on physical fitness

Of the 13 articles on the physical fitness of badminton players, 5 articles related to strength [83, 86, 88–90], 4 articles measured power [83, 86, 89, 90], 5 articles involved speed [83, 85, 86, 89, 90], 2 articles addressed agility [79, 81], 3 articles discussed balance [79–81], and 3 articles examined stability [27, 82, 88].

#### Strength

Of the 13 studies included in this systematic review and meta-analysis, 5 investigated the effects of CST on muscle strength [83, 86, 88–90]. The muscular strength tests employed in these studies encompassed the standing long jump [83, 86, 89, 90], 30-second double shake rope skipping [83, 86, 90], standing shuttlecock kicking [89], one-minute sit-ups [89], 60 seconds push-ups [86], the plank [86], throwing a solid ball from a stationary position, and the shot put throw sideways from a stationary position with 60° and 180° muscle strength tests [83]. Regarding the test results, 3 articles reported an increase in muscle strength following CST [83, 88], 2 articles found no significant difference in muscle strength after CST compared to the control group [89, 90], and the results were mixed in 1 article [86]. Consequently, the impact of CST on muscle strength has yet to be conclusively determined.

#### Power

**Fig 3:**
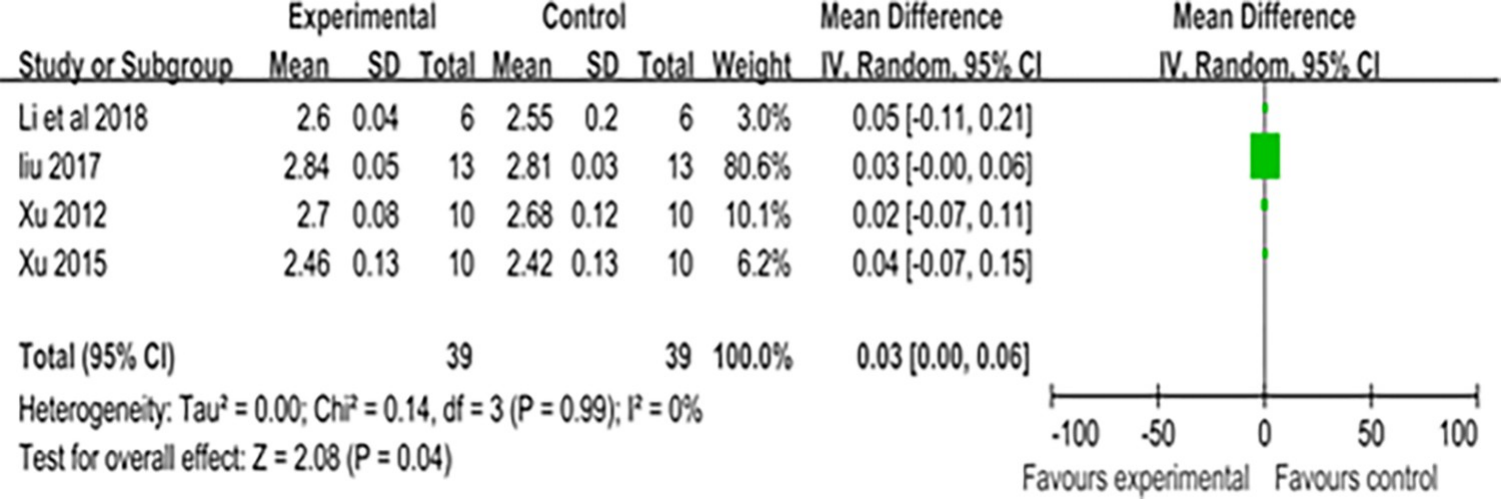
Forest map of influence of CST on standing long jump.

The standing long jump (SLJ) is commonly used to assess lower body explosive power [92–94], and one study has also confirmed the effectiveness of the SLJ for assessing muscle strength or explosive power [94]. This review and meta-analysis include four articles that measure power using the SLJ [83, 86, 89, 90]. A general forest plot for measures of CST and the Standing Long Jump is presented in Fig 3, and the results suggest that CST yields positive effects in badminton players for their muscular strength or explosive power (mean SMD = 0.03, I^2^ = 0%, Chi^2^ = 0.14, df = 2, P = 0.04). According to the results of Egger’s test (P = 0.542) presented in S1 Fig, the four studies exhibit very low heterogeneity.

#### Speed

Speed was assessed with a value of 5 in three of the nine studies included in this review and meta-analysis [83, 85, 86, 89, 90]. The evaluation tools and aspects considered included low center of gravity overturning [89, 90], running with a straight back [90], the 25*5 Shuttle run [89], a quick 40-meter run [86], approximately 5 round trips [86], a 30-meter start run [83], a 25m × 5 round trip run [83], and field moving speed [85]. The results of one article reveal that CST can improve movement speed. However, four articles did not reach a consensus on the measurement of speed.

#### Endurance

Two studies related to muscle endurance employed the core muscle endurance test as their measurement method [79, 80]. One study demonstrated that participants increased muscle endurance through CST [79], while the other found no significant difference in muscle endurance between CST and reinforcement training [80].

#### Balance

Of the 13 studies in this review and meta-analysis, only three focused on balance [79–81]. The Star Excursion Balance Test (SEBT) was utilized in these studies [79–81]. Two of the studies reported that participants’ dynamic balance abilities were enhanced following CST [79, 80]. However, in comparing the CST group with the enhanced training group, both showed improvements in dynamic stability, but no significant difference was observed between them [81].

#### Agility

In this review and meta-analysis, only two of the 13 studies focused on agility [79, 81]. Both studies employed the Illinois Agility Test (IAT) but reported differing outcomes. One study found no significant difference in agility between participants who underwent CST and those who did not [79]. However, the other study indicated that both CST and plyometric training (PT) improved athletes’ agility, with no discernible difference between the CST and PT groups [81].

#### Stability

There are three studies related to stability [27, 82, 88]. Two studies employed the Functional Movement Screen (FMS) scores test method [27, 82], and one used the Biering-Sørensen Test for Muscular Endurance (BFMC) stability testing method [88]. The two studies reported no significant differences in linear squat performance and thematic stability in the Core Stability Training (CST) group compared with the control group (P > 0.05) [27, 82]. Another study showed that a combination of dynamic and static CST effectively improves trunk stability [88]. All three studies demonstrated that CST could enhance stability [27, 82, 88].

#### Effect of core strength training on skill performance

Of the 8 articles on the skill performance of badminton players, 7 involve in-situ skills [27, 82, 83, 86, 87, 89, 90], and there was only one study on move-and-hit skills [84].

#### In-situ skills

Among the 7 articles examining the effect of CST on in-situ skills [27, 82, 83, 86, 87, 89, 90], 4 studies focused on front court skills [27, 83, 86, 89], another 4 on back court skills [83, 87, 89, 90], and only one study addressed midfield skills [83]. The test indicators for front court skills included the spinning net shot [86, 89, 90], net lift [83, 86, 89, 90], push shot [83, 86], crosscourt shot [86], and Lift shot [83, 86]. One researcher found that CST had no significant effect on front court skills [85], while findings from the other three studies were mixed [83, 86, 89]. In terms of midfield skills, some studies have shown that CST positively affects driving skills [87], but three other studies provided inconclusive results on various backcourt skill tests [83, 89, 90]. It is noteworthy that none of the studies compared the impact of CST on different aspects of badminton players’ skills in varying court positions. Additionally, the research on front and back court skills differs, with four studies focusing on net lift skills in the front court and high clear skills in the back court; three of these studies were included in a meta-analysis to verify the findings.

**Fig 4:**
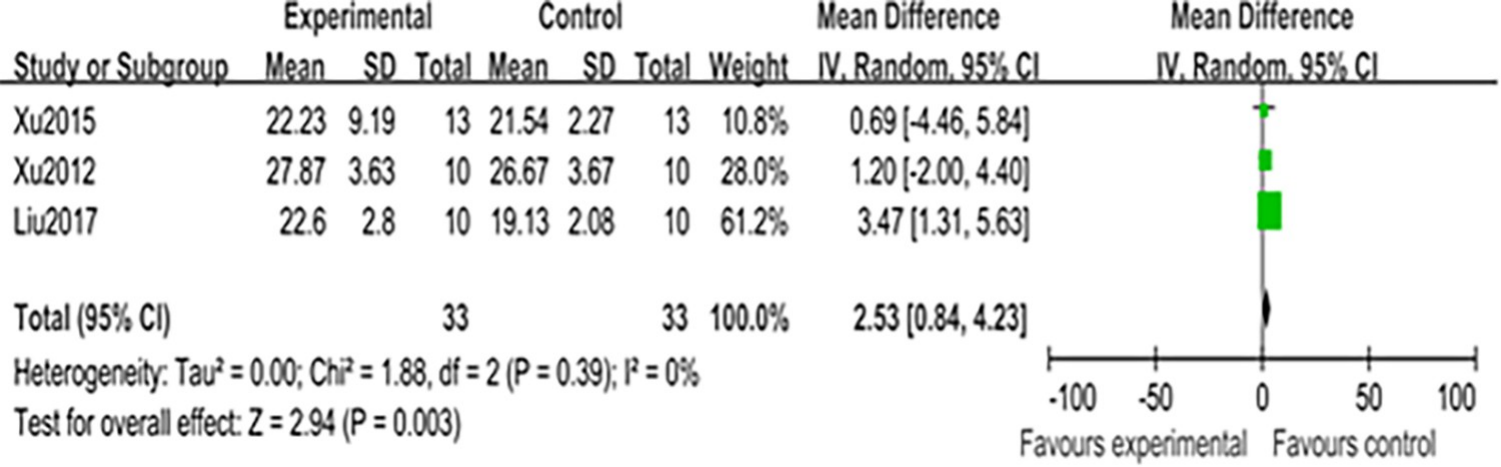
Forest map of influence of CST on net lift.

A meta-analysis of the net lift skills among front-court skills was conducted across three studies [83, 86, 89]. CST was found to have a large effect on badminton players’ performance, with a mean standardized mean difference (SMD) of 2.53 (I^2^ = 0%, Chi^2^ = 1.88, df = 2, P = 0.003) as shown in Fig 4. This effect was significant when compared with other front-court net lift skills. Additionally, the results of Egger’s test, shown in S2 Fig, indicated minimal heterogeneity among these studies, with P = 0.252 (> 0.05).

**Fig 5:**
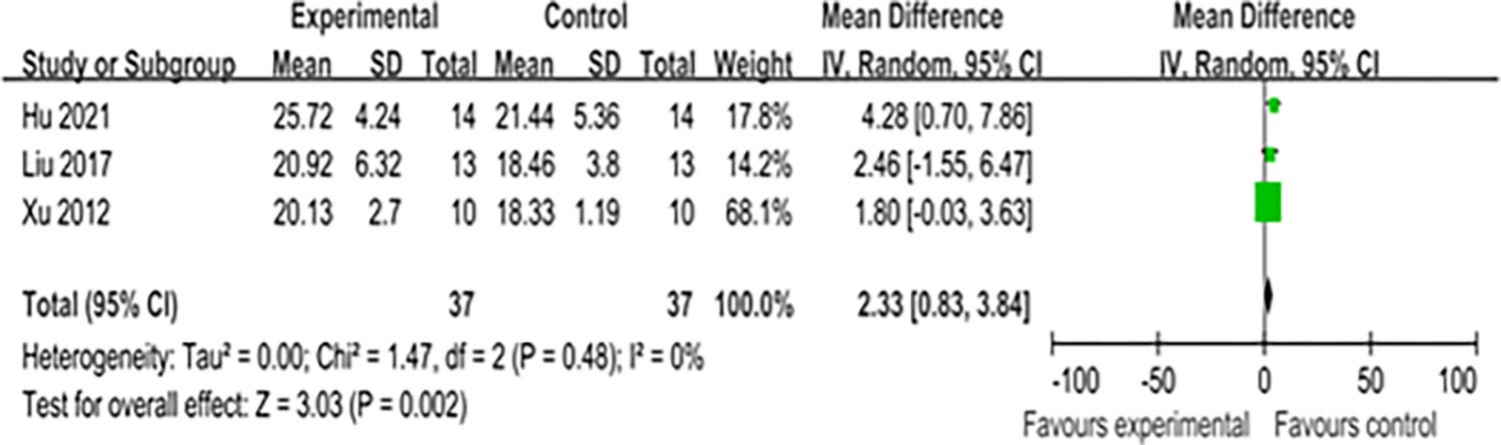
Forest map of influence of CST on hight clear.

A meta-analysis of the apparent height in backcourt skills, encompassing three studies [83, 87, 89], revealed that CST significantly improved badminton players’ performance, with a mean standardized mean difference (SMD) of 2.33 (I^2^ = 0%, Chi^2^ = 1.47, df = 2, P = 0.002) as shown in Fig 5. The results of Egger’s test, shown in S3 Fig, indicated minimal heterogeneity among these studies, with P = 0.734 (> 0.05).

#### Move-and-hit skills

Only one article has studied the move-position-hit skill [84], with the primary indicators tested in this study being straight-line high net shots, full pace movement, jumping smashes, and backcourt two-point lobs. After testing, it was demonstrated that CST had a positive effect on all test indicators.

## Discussion

It is generally believed that core training plays a significant role in enhancing performance and preventing injuries [95]. However, our search did not uncover any research on the effect of CST on injury prevention among badminton players. This review and meta-analysis demonstrate that CST has a positive impact on muscle power, stability, balance, in-situ skills, and move-position hitting skills. Although the benefits for center field skills and move-position hitting skills are recognized and have a positive impact, the existing research lacks sufficient evidence, and there is a need for more authoritative studies. Drawing from 13 pieces of literature, this study primarily explores the effects of CST on the physical fitness and skills of badminton players. On the one hand, appropriate CST can enhance certain aspects of physical fitness. On the other hand, CST positively influences the effectiveness and stability of badminton players’ skills [79].

### Effect of core strength training on physical fitness

Studies have confirmed that CST positively impacts the stability and balance of badminton players [27, 82, 88]. This effect may be attributed to core strength, which involves the muscles’ ability to generate force through contractility and internal abdominal pressure [43]. It also refers to the capacity of both passive and active lumbopelvic stabilizers to maintain reliable torso and hip posture, as well as stability and control, during both static and dynamic motion [9]. Consequently, badminton players can enhance their stability through CST. Regarding dynamic balance, three studies have supported CST’s role in improving badminton players’ balance [79–81], indicating that CST is effective in enhancing balance. This finding is consistent with previous research on balance performance and the activation characteristics of CST [22], further solidifying its status as an intervention to improve balance [96].

We employed meta-analysis to quantitatively analyze long jump results, demonstrating that CST impacts badminton players’ explosive power positively. This improvement is likely primarily due to enhanced neural movement coordination [97]. Notably, the assessment of badminton players’ explosive power, prior to developing effective biomechanical techniques, utilized the standing long jump [83, 86, 89, 90]. Furthermore, our search uncovered additional studies indicating CST’s beneficial effects on explosive power. In one study, 28 athletes were randomly divided into two groups, with both continuing their regular training for eight weeks during the intervention period. Unlike the control group, the experimental group performed a specific action 20 times per session, across three sessions with one minute of rest between each session. After the 8-week intervention, significant improvements in explosive performance were observed in participants from both groups [98].

There is currently no consensus on the effect of CST on badminton players’ muscular strength, speed, endurance, and agility, despite numerous studies underscoring the importance of understanding CST’s impact on these attributes. According to this study, the varied outcomes of CST among badminton players in terms of muscle strength, speed, endurance, and agility may stem from differences in testing methods and the durations of interventions. For instance, in muscle strength testing, a one-minute sit-up test primarily assesses abdominal muscle strength [89], a 60-second push-up test focuses on the chest muscles and triceps [86], and the test involving throwing a solid ball from a stationary position evaluates the coordination and integration of the whole body’s muscles [83]. There was no observed change in agility test results between the experimental and control groups of badminton players after six weeks of CST conducted twice weekly. However, significant improvements in agility test scores were noted for both groups following four weeks of CST conducted five times weekly [79, 81].

The impact of CST on the flexibility and coordination of badminton players represents a research gap. However, flexibility and coordination are crucial for badminton players; for example, players need to be flexible to effectively employ the badminton smash [99]. Additionally, the level of coordination badminton players possess significantly influences their performance [100]. Therefore, future research should investigate how CST affects badminton players’ coordination and flexibility.

### Effect of core strength training on skill performance

Among the effects of CST on in-situ skills, only one study demonstrated a positive effect of CST on midfield skills [83], primarily focusing on drive skills. The diverse research goals of the designers are expected to yield different results. Notably, 7 articles examined the effect of CST on in-situ skills [27, 82, 83, 86, 87, 89, 90]. Four studies focus on front court skills [27, 83, 86, 89], including spinning net shots, lift shots, crosscourt shots, and push shots. Another four studies address backcourt skills [83, 87, 89, 90], featuring drop shots, high clears, and smash skills. The meta-analysis confirms that CST has a positive impact on both badminton’s front court and in-situ backcourt skills. Thus, this study verifies that CST beneficially influences badminton players’ situational skills. Although there is no consensus on specific badminton test skills, this review and meta-analysis suggest that the selection of skills for research is contingent upon the researcher’s objectives, with different objectives leading to varied research designs.

The researchers found that badminton players significantly progressed in in-situ and on-move position hit skills after undergoing CST. This improvement may be attributed to CST’s ability to enhance the nervous system’s coordination of muscle groups, thereby improving movement efficiency and the skill performance of badminton players [9]. Hassan suggests that the positive effects of CST on the skill of test subjects could be explained by the core’s role in the movement chain and the fact that skill acquisition depends on several factors, including lower body muscular strength, leg power, skill, and the correct kinetic chain [9, 48]. Low-intensity static core stability training, which serves as the foundation of the entire training plan, must be incorporated into core training [43]. Meanwhile, high-load dynamic CST leads to hypertrophy of muscle fibers and an increase in muscle strength [101]. The proactive role of CST in the movement chain enhances badminton players’ skills. Another factor contributing to badminton players’ skill improvement could be their enhanced stride through CST. The stride and shuttlecock movements are closely linked, with joint contact force playing a crucial role in this action. Joint contact force, the actual force exerted on the articular surface, may predict both performance and the risk of injury [102]. An intense impact occurs at heel contact during a badminton player’s repeated lunge steps [103]. Core strength significantly affects an athlete’s ability to generate and transfer forces [104]. Athletes can perform better and find it easier to respond to the shuttlecock with CST, as it provides greater control over landing on one or two feet.

A significant gap was identified across the 13 studies, with none examining a single skill across different states, such as the badminton spike, which is one of the most powerful skills in all racket sports [105]. Empirical evidence suggests that adjusting the body’s position relative to the incoming shuttlecock is crucial for producing a powerful and accurate spike [106]. However, the research has mainly focused on the badminton spot smash, which varies across different states. Moreover, only one article examined the move position hit skills [84], indicating a need for further research in this area. For instance, skills such as the smash, high clear, drive, spinning net, crosscourt, push, and lift shot have not been thoroughly studied. Badminton is currently the second most popular sport worldwide after football, with an estimated 220 million people playing it regularly, from professional to recreational levels [107, 108]. Unfortunately, this study, through a literature review, shows that scientific research on badminton skills is less common than might be expected for such a popular sport [108]. There is a need to continue strengthening CST research to further understand its impact on badminton skill-related areas.

## Limitations

Overall, this review and meta-analysis of 13 studies provide evidence that Core Stability Training (CST) positively impacts badminton players’ muscle strength, stability, balance, in-situ skills, and move-position hit skills. However, the review has several shortcomings, primarily in the areas listed below:

1. Among the 13 studies, researchers used different types of tests. When differences occurred in the studies, common indicators could only be used to find answers through meta-analysis.

2. Different training cycles, training times, and training frequencies of CST may impact athletes’ physical fitness and technical performance, but it is not easy to reach a consensus because the data is too scattered, and the emphasis of various researchers is different.

3. Due to the limited articles on the effect of CST on badminton players’ performance, only a meta-analysis was conducted on the three common research indicators of researchers.

4. This review covers studies where the paper did not specifically look at female athletes, which limits our understanding of CST’s general efficacy in improving badminton players’ performance.

5. Because the researchers’ design schemes vary, we cannot perform additional PT frequency, length, and training time analyses. Therefore, we cannot give definitive advice on the optimal intensity of badminton players’ performance.

6. Since only one researcher used dynamic CST and static CST, and other researchers used combined CST, comparing the training effects of dynamic CST and static CS on badminton players is impossible.

## Conclusions

Through CST, badminton players of all ages can improve various aspects of their physical fitness, including muscle strength, power, balance, and stability, as well as in-situ skills (front-court and back-court skills) and move-position hit skills. However, the impact of CST on players’ speed, endurance, and agility has not been conclusively determined. Furthermore, the effect of CST on the flexibility and coordination of badminton players represents a significant research gap. Specifically, the influence of CST on badminton players’ physical fitness, particularly flexibility, and move-position hit skills (such as smash, high clear, drive, spinning net shot, crosscourt shot, push shot, and lift shot skills), has not been adequately studied, with a noticeable lack of research on female athletes. Therefore, further research into the impact of CST on the physical and technical performance of female athletes is warranted.

## Supporting information

S1 Checklist (DOCX)

S1 Fig Egger’s test of CST on standing long jump. (PNG)

S2 Fig Egger’s test of CST on net lift. (PNG)

S3 Fig Egger’s test of CST on hight clear. (PNG)

S1 File (XLSX)
