# Supplementary figures and images for: Effect of core strength training on the badminton player’s performance: A systematic review & meta-analysis
Source: PLoS One. 2024 Jun 12;19(6):e0305116. doi: 10.1371/journal.pone.0305116 (PMC11168634; doi:10.1371/journal.pone.0305116)

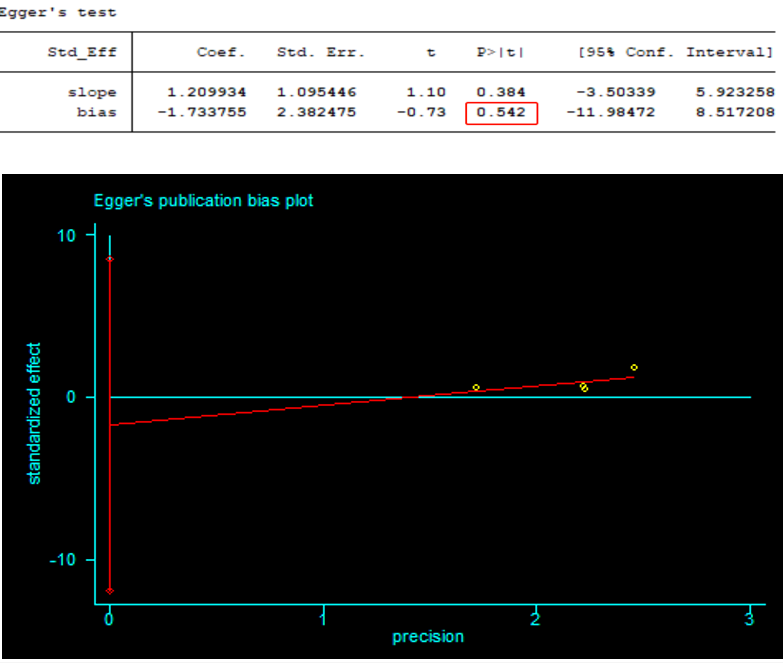

Supplement: S1 Fig — (PNG) [file pone.0305116.s002.png]

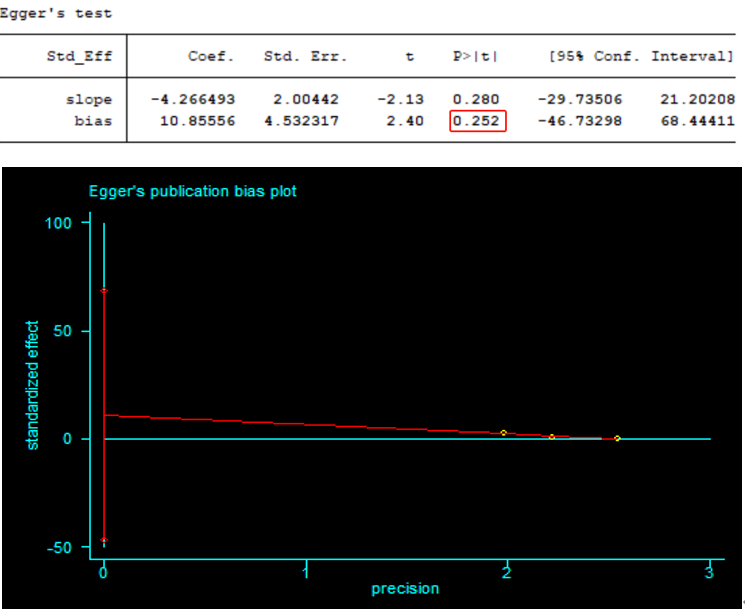

Supplement: S2 Fig — (PNG) [file pone.0305116.s003.png]

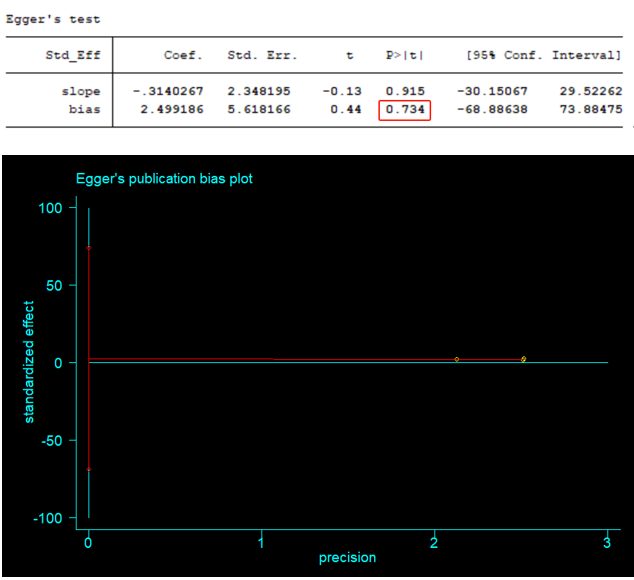

Supplement: S3 Fig — (PNG) [file pone.0305116.s004.png]
